# Effects of oral contraceptives on spatial cognition depend on pharmacological properties and phase of the contraceptive cycle

**DOI:** 10.3389/fendo.2022.888510

**Published:** 2022-09-06

**Authors:** Elizabeth Hampson, Erin E. Morley, Kelly L. Evans, Cathleen Fleury

**Affiliations:** ^1^ Department of Psychology, University of Western Ontario, London, ON, Canada; ^2^ Schulich School of Medicine and Dentistry, University of Western Ontario, London, ON, Canada

**Keywords:** oral contraceptive, hormonal contraceptive, menstrual cycle, estrogen, androgen, ethinyl estradiol, mental rotation, visuospatial

## Abstract

The central nervous system effects of oral contraceptives (OCs) are not well-documented. In a set of 3 studies, we investigated a specific cognitive function, mental rotation, in healthy women currently using OCs for contraceptive purposes (*n* = 201) and in medication-free controls not using OCs (*n* = 44). Mental rotation was measured using a well-standardized and extensively validated psychometric test, the Vandenberg Mental Rotations Test (MRT). In an initial study (Study 1), current OC users (*n* = 63) were tested during the active or inactive phases of the contraceptive cycle in a parallel-groups design. Studies 2 and 3 were based on an archival dataset (*n* = 201 current OC users) that consisted of data on the MRT collected in real-time over a 30-year period and compiled for purposes of the present work. The OCs were combined formulations containing ethinyl estradiol (10-35 ug/day) plus a synthetic progestin. All 4 families of synthetic progestins historically used in OCs were represented in the dataset. Cognitive performance was evaluated during either active OC use (‘active phase’) or during the washout week of the contraceptive cycle (‘inactive phase’) when OC steroids are not used. The results showed a significant phase-of-cycle (POC) effect. Accuracy on the MRT was mildly diminished during the active phase of OC use, while scores on verbal fluency and speeded motor tasks were modestly improved. The POC effect was most evident in women using OCs that contained first- or second-generation progestins (the estrane family of progestins or OCs containing levonorgestrel), but not in women using OCs containing recently developed progestins and lower doses of ethinyl estradiol. Using independently established ratings of the estrogenic, androgenic, and progestogenic intensities of the different OC formulations, each brand of OC was classified according to its distinct endocrine profile. Multiple regression revealed that the effects of OC use on the MRT could be predicted based on the estrogenic strength of the contraceptives used. Estrogenic potency, not androgenic or anti-androgenic effects of the OC pill, may underlie the effects of OC usage on spatial cognition.

## 1 Introduction

Oral contraceptives (OCs) have been available since the 1960s as a trusted method of contraception and are used by millions of women world-wide. Most contemporary OCs are ‘combined’ formulations that consist of an orally administered estrogen (typically ethinyl estradiol, EE2) in combination with a progestin. At present, more than 20 different synthetic progestins are available for contraceptive use. Different brands of OCs are differentiated by which progestins they contain, their pharmacological properties, and the dosages of estrogen and progestin that are used. Despite widespread adoption by women, scientific knowledge of OC actions in the central nervous system (CNS) is still rudimentary. In recent years, there have been multiple calls for increased study of the CNS effects of OCs (1, 2).

OCs disrupt the endocrine environment of the female menstrual cycle. Through feedback inhibition of the hypothalamic-pituitary-gonadal axis, OCs inhibit gonadotropin secretion and the rising concentrations of endogenous 17β-estradiol that trigger ovulation (3). Because ovulation is prevented, the luteal phase increase in progesterone that normally follows ovulation does not occur. Circulating testosterone is reduced by 40-60%, as revealed by assays of free testosterone in the serum or saliva of OC users (e.g., 4, 5). While endogenous production of sex steroids is inhibited, OCs serve as an *exogenous* source of estrogens and progestins, whose resulting levels in the bloodstream vary depending on the brand of OC used and individual differences in metabolism and clearance (6, 7; but see 8). While standard commercial immunoassays of serum or saliva confirm the lower levels of endogenous hormones, they are typically insensitive to the exogenous hormones contained in OC pills, rendering the exogenous hormone ‘invisible’ in standard laboratory assays. Importantly, however, the OC steroids can interact physiologically with hormone receptors located in the CNS (and elsewhere in the body) and thus possess a potential to influence CNS activity and function (e.g., 9, 10). Indeed, recent studies employing advanced neuroimaging techniques such as functional MRI, while subject to methodological limitations, suggest OC use may be associated with subtle changes in brain structure and resting-state and/or task-driven neuronal activity (see 11 for review). Changes have been identified in several different brain regions, including hippocampal and neocortical sites known to participate in higher-order cognitive processes (11, 12).

Although empirical data are still sparse, OCs might plausibly be expected to influence certain spheres of cognitive function. Furthermore, the effects are likely to be selective. Over the past 3 decades, the naturally-occurring forms of estradiol and progesterone, which vary over the menstrual cycle in healthy *non*-OC users, have been discovered to modify certain cognitive functions, notably those that are sex-differentiated (i.e., display sex-related differences in performance). These effects are mediated by the binding of ovarian steroids available in the bloodstream to estrogen-, androgen-, or progesterone receptors in the CNS (13, 14). Local alterations in neurochemistry or synaptic function are then initiated *via* modulatory effects of the steroids on gene transcription or *via* rapid non-genomic signaling mechanisms (14, 15). While evidence is not entirely consistent, high levels of 17β-estradiol, the major estrogen present in naturally-cycling women of reproductive age, have been associated with modest increases in verbal fluency, perceptual speed and accuracy, and possibly verbal memory (e.g., 16–18), but also diminished performance on tests of visuospatial ability including mental rotation tests (e.g., 16, 19–21). One question, therefore, is what effect does the use of *oral contraceptives* have on women’s cognition? Secondly, are the effects of OCs attributable to their suppression of the endogenous steroids? Or to the exogenous hormones supplied by the OCs themselves? Based on precedent established by studies investigating natural forms of the hormones, visuospatial cognition is one possible candidate to exhibit an OC-linked effect.

**Figure 1:**
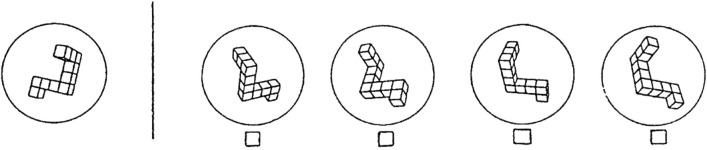
An example item from a task that requires mental rotation, the ability to ‘rotate’ an object in one’s mind. This item is from the Vandenberg Mental Rotations Test (MRT), a psychometric tool developed and standardized by Vandenberg and Kuse (31). Each of the 24 items on the test depicts a target object (left) that can be rotated to match only two of the multiple-choice options shown on the right. Which two are correct? Mental rotation is an elemental process involved in visuospatial cognition. [Reprinted from *Hormones and Behavior*, Vol. 65, Hampson E, Levy-Cooperman N, Korman JM, Estradiol and mental rotation: Relation to dimensionality, difficulty, or angular disparity? pp. 238-248, 2014, with permission from Elsevier].

To date, studies of cognition and perception in women using OCs are few in number. Possible effects of OCs on a range of outcomes have been suggested, from olfactory and inner ear processes (4, 22, 23), to motor execution or planning (24), memory processes (25–28) and controversially, vulnerability to mood disorders (e.g., 29). Empirical support for such effects, however, is limited and unsystematic. Within the realm of cognitive function, a handful of studies has focused on visuospatial cognition, defined as the capacity to envision in the ‘mind’s eye’ the visual appearance, positions, or movements of objects (30). One common example of a visuospatial ability is mental rotation, i.e. imagining the movement of an object around its axis or envisioning the orientation of a rotated object when viewed from different vantage points (see **Figure 1**).

Existing studies of OCs and mental rotation have produced inconsistent findings, are typically limited to comparisons of naturally-cycling women (NC) and mixed groups of OC users (often without controlling for the phase of the menstrual cycle where cognitive testing is performed), and are subject to a range of methodological issues. These include small sample sizes that render it difficult to draw conclusions, particularly given the diversity of OC formulations available. On average, cognitive studies have included a mean sample size of only 24 OC users (32). The earliest work on mental rotation suggested no differences exist between OC users and non-users, with both groups of women however showing improvement in accuracy if tested during the menstrual phase of their menstrual cycle (33, 34). Other studies, to the contrary, have suggested that active use of OCs might improve mental rotation slightly compared with non-OC users (e.g., 17, 35, 36), or that either improved or diminished performance can be found depending on the ‘androgenicity’ (or ‘anti-androgenicity’) of the progestin constituent of OCs, with superior visuospatial ability observed under conditions where an OC contains a progestin having a greater capacity to bind to androgen receptors (37). Of note, some but not all of the progestins used in OCs are derived from 19-nortestosterone and exhibit residual binding affinity for androgen receptors. On the other hand, our own past work has suggested that visuospatial performance may be linked to the estrogenic, not androgenic, effects of the OC pill (35, 38).

Despite early publication barriers, our laboratory has systematically collected data over many years to investigate the effects of natural and synthetic reproductive steroids on cognitive function, perceptual processes, and motivational variables in both OC users and non-users. Relevant to the present report, we have collected data over the past 3 decades using the Vandenberg test of mental rotation (MRT; 31) to study visuospatial function. Vandenberg’s test is a modification of the Shepard-Metzler (39) figures and is a ‘paradigmatic’ index of visuospatial ability. We first began collecting data on the MRT in 1991 as part of our wider body of work on the modulation of visuospatial abilities by endogenous estradiol in women. The resulting data from non-OC users has been published (e.g., 21, 40), but much of our OC data (often collected either as control groups or to assure that cognitive testing was conducted blind to women’s endocrine status) has not. In the present article we report previously unpublished findings from a historical dataset of OC users, compiled *via* a series of real-time studies carried out in our laboratory between 1991 and 2015. These data were collected by former students in the laboratory and together comprise an unreported dataset of significant informational value because the number of OC users is unusually large (*N* = 201), it consists of healthy young adults using OCs strictly for contraceptive purposes (absent of pre-existing medical conditions) who were tested on a well-validated, widely-used, cognitive test (the MRT; 31) collected in real time over multiple ‘generations’ of the synthetic progestins used in standard OCs. Data throughout this 30-year period were collected by several different experimenters but used identical test stimuli, identical test administration procedures (the 24-item version of the MRT, administered using a 4-minute time limit for each half of the test), and identical scoring criteria (31; see details below). All assessments were carried out under well-controlled, in-person, one-on-one test sessions in our laboratory. Internet testing was avoided. To our knowledge, it constitutes the largest dataset ever collected on mental rotation in women who use OCs and every ‘generation’ of contraceptive progestin, from the first to most recent, is represented. As such, the dataset is a rare, possibly unique, resource to test the general hypothesis that OCs influence cognitive function, and the specific hypothesis that the use of OCs is associated with hormonally-driven effects on mental rotation.

The purpose of the present report is to explore whether OCs influence higher-order cognitive processes, using mental rotation as a target function. We aimed to test whether any OC effect could be identified; to explore whether the effect differs across different families of OCs; and to investigate if a phase-of-cycle effect is associated with OC use, as is demonstrated across the natural menstrual cycle in naturally-cycling women (18–21). A phase-of-cycle effect has not been established in OC users to date. This question, however, is important theoretically because it addresses the unresolved issue of whether cognitive effects of OCs are caused by the exogenous steroids present in OC pills or by their suppression of endogenous 17β-estradiol or the naturally-occurring progestogen, progesterone. To address these questions, we first report (in Study 1) the results of a medium-sized investigation of OC users and non-user controls who were tested on a set of sex-differentiated cognitive tasks while actively taking their OCs or during the ‘off’ week of the contraceptive cycle (when no hormone is used and therefore concentrations of exogenous hormones are minimized). In Study 2, we then examine the hypothesis of phase-of-cycle effects across all 4 families of synthetic progestins (explained further below) using our full dataset of 200 OC users. Finally, in Study 3, we end by investigating whether observed effects of OCs on mental rotation are related to the estrogenic or androgenic actions of OC pills, and which hormonal constituent is most important in driving the mental rotation effect. This question is explored in Study 3 *via* multiple regression methods.

## 2 Study 1: the ‘on-off’ study

### 2.1 Background and hypothesis

In naturally-cycling (NC) women who do not use OCs, accuracy on mental rotation tests shows small but systematic variations across the menstrual cycle (for a recent review see 41). Scores on the MRT tend to improve during menses, the phase of the menstrual cycle when circulating levels of ovarian steroids are lowest, and are modestly diminished at phases where 17β-estradiol concentrations are at their peak (e.g., 18–21, 33, 42). Conversely, high estradiol is associated with slight improvements in performance on certain motor planning/execution tasks and in verbal fluency (16, 18). (Verbal production is assessed in the laboratory by having participants generate words, phrases, or sentences that meet experimenter-defined semantic, lexical, or phonetic criteria). At lower levels of estradiol increase, menstrual cycle effects are harder to detect (e.g., 43), consistent with the relatively small effect sizes of these hormonal influences.

In women using OCs, two early studies (33, 34) raised the possibility that a similar phase-of-cycle (POC) effect might be detectable among OC users. However, these early findings were largely dismissed because of methodological concerns (e.g., failing to analyze OC users separately from non-users in statistical analyses; inconsistent findings). As a result, POC effects among OC users remain unconfirmed. A few later studies that did try to address the POC question were complicated by practice effects that rendered the findings inconclusive (e.g., 26, 44, 45). While generally recognized as more statistically powerful, within-subject designs are not recommended in situations where differential carryover effects can be anticipated (46). The MRT is known to elicit a substantial practice effect (47) and to have decreased validity with multiple test exposures. To avoid the complications caused by repeat testing, therefore, we used a between-subjects design to test the hypothesis of a POC effect.

Based on past investigations of NC women, we hypothesized that a POC effect (operationalized here as a difference in performance between the active and inactive phases of the contraceptive cycle) would likewise be found in OC users if the exogenous hormones contained in OCs are responsible, because tissue concentrations of OC steroids are higher during the active phase of the OC cycle (when hormones are ingested on a daily basis) than during the inactive phase (when no hormone is used, resulting in washout of exogenous hormones and onset of menses). Conversely, the suppression of endogenous hormone production caused by OC use endures during the inactive phase of the contraceptive cycle (e.g., 26, 44) and may even last for weeks in some women. In particular, 17β-estradiol and progesterone levels either remain stable during the inactive phase or show a minute rise only, approximating early follicular values (e.g., 26, 48, 49). Consequently, no differences in MRT performance were anticipated between the active and inactive phases if suppression of endogenous hormones is the mechanism responsible for cognitive differences between OC users and non-users. Importantly, following past observations in NC women, we predicted that any cognitive effects seen in OC users would be directionally-selective, and that the direction of effect would depend on the precise cognitive processing requirements demanded by the tasks performed.

### 2.2 Methods

#### 2.2.1 Participants

OC users (*N* = 63) were recruited *via* poster advertisements at a Canadian university and reimbursed for their participation (includes two participants with partial data only). All participants were right-handed and had been using the same brand of OC for a minimum of 4 mo prior to participating in the study. Only women using ‘low-dose’ OCs were included (30-35 ug EE2 per day). As the dataset is historic, all participants were taking first or second generation progestins (see **Supplementary Materials Table S1**, for a full list of the OC brands used). Volunteers were pre-screened by telephone interview to assure that the inclusionary and exclusionary criteria were met. Participants were considered ineligible if they had been using OCs for less than 4 mo, were left-handed, had any history of psychiatric, endocrine, or neurological conditions, or if they used any prescription medication other than OCs.

Data from a demographically-matched group of NC women (*N* = 44), recruited in the same manner and tested on the MRT, were also included. The NC group was recruited from the same source and were matched on educational level and within 1.5 years of age. The NC women were tested at either menses, when ovarian output is negligible (corresponding to the ‘inactive’ phase), or at higher estrogen (‘active’) phases of their menstrual cycle, as confirmed by salivary radioimmunoassays of 17β-estradiol and progesterone collected at the study visit (see 21 for full details). The purpose of the NC group was to provide a context for the MRT scores observed in the OC users, by illustrating a typical level of performance in demographically-matched NC controls, who had been tested at 2 phases of the natural ovarian cycle found in past work to differ in their average level of performance on the MRT (e.g., 18, 20). Mean age was 22.17 ± 3.25 years (SD).

#### 2.2.2 Procedure

The study employed a between-subjects design to avoid potential confounding introduced by repeated exposure to the cognitive test materials. Each participant reported individually to our lab for a 60-min study visit where a brief set of verbal and spatial cognitive tasks and simple motor tasks were administered by a trained examiner. Participants also filled out a standardized mood scale (Profile of Mood States, 50), a handedness inventory (51), and an auditory test (not relevant to the present report).

Based on information collected during telephone pre-screening, half the women were scheduled to be tested during the inactive phase of the OC cycle (Days 5, 6, or 7 of the 7-day inactive phase) when no hormone is taken, and half were tested during the 21-day active phase when OC pills containing active EE2 plus a progestin are taken. Assignment to phase was counterbalanced. Because it can take several days for exogenous hormone concentrations to stabilize at the beginning of a new OC package (6, 7, 52), women at the active phase were tested after at least 5 days of active pill use and, if using a multiphasic brand of OC (with varying hormone levels) were tested after taking at least 2 pills containing the highest hormone content. The average number of active pills remaining in the present package on the day that cognitive testing was performed was 7.45 pills (*SD* = 4.14). Participants were required to bring their current package of OC pills to the study visit to allow the researchers to objectively verify the name and type of OC used, the prescription information, and to verify the exact number of pills remaining on the date of cognitive testing.

#### 2.2.3 Cognitive tests

Five cognitive tests were administered. Detailed descriptions can be found in **Supplementary Table S2**. These tasks were selected because they, or highly similar tests, have exhibited sensitivity to circulating levels of reproductive hormones in past studies of the natural menstrual cycle. Visuospatial tests included the Mental Rotations Test (MRT; 31) and the Paper Folding Test (53). The MRT is widely used in laboratory settings to assess proficiency of mental rotation. The 24-item version of the standard MRT was used with a four-minute limit on each half of the test. On each item participants had to identify which two of four forced-choice alternatives were rotated versions of a target object. The only difference between the target object and the two correct objects was their angular orientation in space. The test was scored using the standard scoring criteria recommended by the original test developers (31) and the final score was corrected for guessing (max correct = 48). The second test, Paper Folding (53), assessed a different form of spatial ability. Each of the 20 items requires predicting where punched holes would be located in a folded piece of paper once the paper is unfolded again. The correct answer is identified from among 5 alternatives. Six minutes were allowed. The number correct summed over all 20 items was calculated, and the total score was corrected for random guessing (53).

As seen in **Supplementary Table S2**, the remaining cognitive tasks were non-spatial. They consisted of two verbal fluency tasks, and a simple motor learning task (Manual Sequence Box; 54) that required learning and then executing a sequence of 3 hand postures until a criterion of 10 consecutive sequences without error was reached. In a prior study using a repeated-measures design, we previously reported that this form of motor learning is facilitated during the active phase of the OC cycle, compared with the inactive phase (24). Over the natural menstrual cycle, several independent labs have reported superior verbal fluency during high-estrogen phases compared with low (16, 18), and a correlation between verbal fluency scores and circulating levels of 17β-estradiol has been seen in NC women (18, 55–57).

#### 2.2.4 Statistical analysis

Scores on the MRT were analyzed using a univariate ANOVA with Contraceptive Status (OC or NC) and Phase-of-Cycle (active, inactive) as independent variables. Because the NC women were originally recruited for a separate study, they overlapped with the OC group only for the MRT, but received different verbal fluency tasks and therefore could not be included in the statistical analyses of the non-spatial measures. Accordingly, the other measures were analyzed for the OC users alone using separate tests (one-tailed *t*-tests) that did not include the NC women.

### 2.3 Results

**Figure 2:**
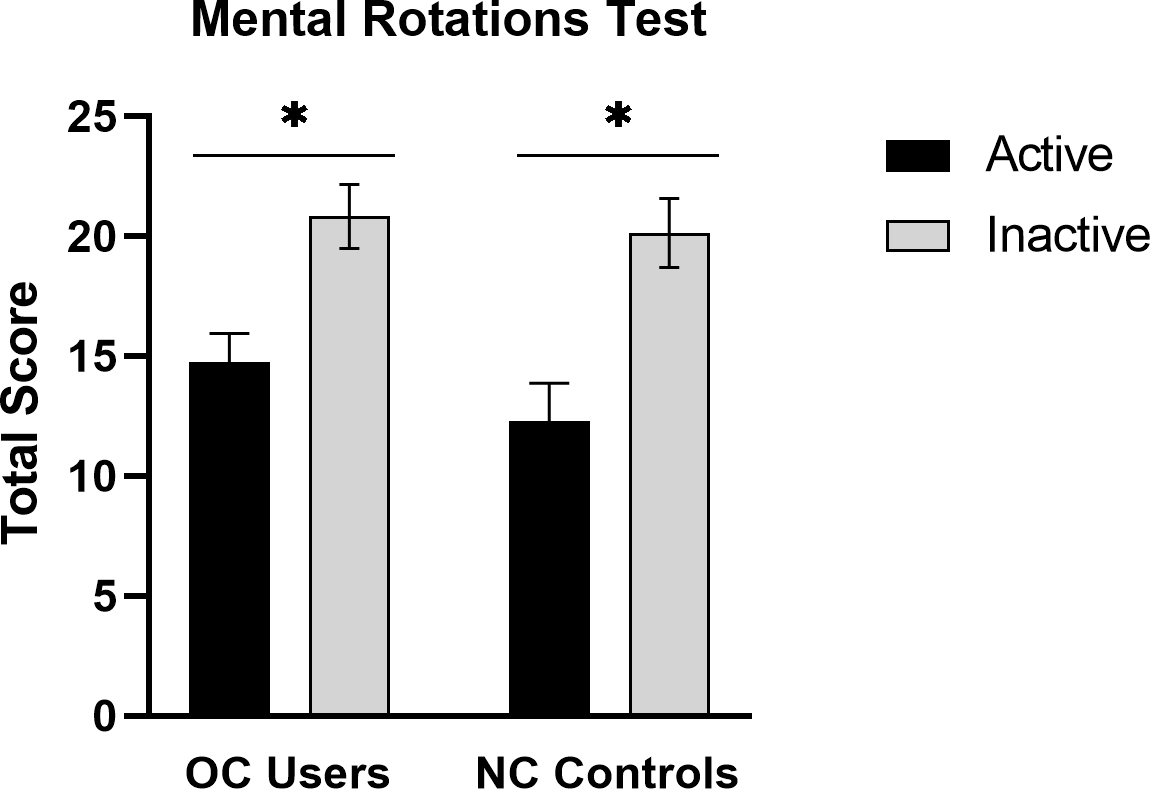
Mean accuracy on the MRT at the active and inactive phases of the menstrual cycle in oral contraceptive users (OC users, n = 61) and naturally-cycling controls (NC controls, n = 44). A robust phase-of-cycle effect was found in both groups. Among OC users, mental rotation performance was superior during the inactive phase of the contraceptive cycle (grey bars), when exogenous hormone intake is absent. *p < .01.

Results for the MRT are shown in **Figure 2**. A robust phase-of-cycle (POC) effect was found. The POC effect was evident in both the NC and OC groups, *F*(1,101) = 24.72, *p* < .001. The interaction between POC and contraceptive status (OC user or non-user) was not significant, *F*(1,101) = 0.38, *p* = .538. The magnitude of the effect, expressed as Cohen’s d (58) was d = 0.84 for OC users and d = 1.14 for the NC controls (see **Figure 2**). As expected, accuracy on the MRT was significantly higher in OC users who were tested during the inactive (menstrual) phase of the OC cycle, compared with OC users tested under ‘active’ hormone conditions. In other words, performance was poorer under active OC use.

The other visuospatial task, Paper Folding, showed the same pattern of means as the MRT (see **Table 1**), and approached significance, *t*(59) = 1.57, *p* = .061. In the present sample, average accuracy on the Paper Folding test was higher during the inactive phase, d = 0.41. The smaller effect size is commensurate with the smaller sex difference that is usually reported for Paper Folding, relative to the MRT task (59).

**Table 1:** Mean performance (SD) on the MRT and other cognitive tasks at the active and inactive phases of the contraceptive cycle.

	Active Phase	Inactive Phase	Cohen’s d	*p*-value
Mental Rotations Test (# correct)	14.72 (5.67)	20.82 (8.50)	d = 0.84	*p* = .001
Paper Folding (# correct)	11.97 (3.67)	13.36 (3.06)	d = 0.41	0.061
Oral Fluency (# words generated)	12.44 (3.53)	10.75 (4.66)	d = -0.41	0.055
Controlled Associations (# words)	30.45 (7.70)	26.82 (7.01)	d = -0.49	0.029
Box Task – time to acquisition (sec)	12.02 (2.42)	14.46 (5.23)	d = -0.60	0.013
Box Task – speeded execution (sec)	14.30 (3.83)	16.95 (5.88)	d = -0.53	0.022

The Manual Sequence Box is a timed measure, therefore a lower score indicates better performance.The symbol # stands for "number" as in "number correct".

A significant but reversed direction of effects was found for the Manual Sequence Box. A significant POC effect was seen, Acquisition: *t*(38.6) = 2.30, *p* = .013; Execution: *t*(47.4) = 2.06, *p* = .022. As expected, OC users displayed better motor performance during the active than inactive phase (**Table 1**). This confirms results reported for the same manual sequencing task by Szekely et al. (24) in an earlier, independent, sample of OC users. Both of the verbal fluency tests likewise showed a significant or near significant POC effect, *p* = .029 for Controlled Associations; *p* = .055 for the Oral Fluency test (**Table 1**). On both fluency tests, word retrieval was superior during the active phase of the OC cycle, when exogenous hormones are used on a daily basis. Thus the direction of the POC effect for the MRT task (superior performance at the *inactive* phase) was reversed on the verbal measures, which depend on separate brain regions and do not recruit visuospatial processes. This suggests the visuospatial effect is selective.

Because the OC group contained women taking both monophasic (*n* = 24) and multiphasic (*n* = 39) OC formulations, a secondary ANOVA was run to test if the MRT results might be attributable to this difference. The results, however, showed no significant difference in accuracy between the women using mono- (*M* = 18.32) or multiphasic OCs (*M* = 17.99), *F*(1,57) = 24.72, *p* = .866. The MRT scores were nearly identical in both subgroups.

### 2.4 Discussion

Study 1 confirmed a significant POC effect among OC users. The direction of the effect under active OC use differed for the MRT versus the verbal tasks, demonstrating selectivity based on function. For all tasks, the direction of effect conformed to the POC effects reported to occur in naturally-cycling women, in association with naturally-occurring variations in ovarian hormones across the menstrual cycle (e.g., 16, 18, 42). In particular, lower accuracy on visuospatial tasks and improved verbal performance has been found in NC women when elevated concentrations of ovarian hormones are available to the CNS.

Study 1 confirmed early reports by Moody (33) or Silverman and Phillips (34) that imply a potential POC effect might exist in OC users (see also 26 for parallel findings on a verbal memory task). Those early studies were inconclusive, reflecting methodological issues that left the findings open to question. Consistent with Moody (33) and Silverman and Phillips (34), all participants in our ON-OFF study used OCs containing 1^st^ or 2^nd^ generation progestins. Thus, while Study 1 demonstrates a POC effect it does not establish whether a POC effect can also be found in women using modern 3^rd^ or 4^th^ generation OCs that contain lower EE2 concentrations and/or progestins possessing different pharmacological properties.

Recent OC studies have overlooked the question of POC effects. This might reflect lack of awareness, or the exclusion of women not at the active phase of the OC cycle in certain studies (e.g., 60, 61), or small sample sizes inadequately powered to detect POC effects. In some studies data are simply collected at random points in the OC cycle and then combined, irrespective of the possibility of POC effects on outcomes (35). Many recent investigations have compared OC users as a whole with NC women (who may or may not be assessed at known points in the natural menstrual cycle) to discover how the performance of OC users might differ from NC controls (e.g., 62). Failure to understand or identify POC effects is potentially an important source of measurement error in OC studies. Claims of ‘better’ or ‘worse’ performance by OC users are not well-substantiated by studies that fail to control for POC effects. If not controlled, apparent differences in accuracy on the MRT between OC and NC groups could merely reflect chance differences in the proportions of women in the 2 groups who happened to be tested during menses. On the other hand, if POC effects are attributable to the exogenous hormones present in OCs, then POC effects conceivably might be less visible for current formulations because they are attenuated by the lowered EE2 doses used in many contemporary OCs or are linked only to specific subclasses of synthetic progestins.

## 3 Study 2: phase of cycle effects across different families of oral contraceptives

### 3.1 Background and hypothesis

The objective of Study 2 was to explore the generalizability of POC effects across the different classes of progestins used in OCs. We analyzed an archival dataset consisting of 201 OC users, to investigate if POC effects are evident when the family of OC pills being used is considered as an independent variable.

The term ‘generation’ is used in 2 different senses in the medical literature. Some researchers define generations based on the timing of an OC’s introduction into the North American marketplace, while others use the term to denote the distinct families of progestins available for use in OCs based on differences in molecular structure (63). We adopt the latter convention here. The progestin families are distinguished by their pharmacological profiles and derivation from either 19-nortestosterone or 17-hydroxyprogesterone or, more recently, spironolactone (64). Endocrine differences between the families include their capacity to exert androgenic or anti-androgenic side-effects, but also their capacity to exert estrogenic or anti-estrogenic effects (or neither), their progestogenic intensity, presence of mineralocorticoid effects, effects on serum binding proteins (e.g., SHBG), and their relative ability to bind to classical steroid hormone receptors (65). The estrane- and early gonane-based OCs used in Study 1 have stronger androgenic effects *in vivo* (but often contain higher doses of EE2) than many OCs based on 3^rd^ or 4^th^ generation progestins (10, 66), some of which have *anti-*androgen effects at concentrations used therapeutically (e.g., drospirenone; 65). One widely-recognized classification of the progestin families, which is based on structural properties of the progestins, is shown in **Table 2** (64, 67), and was used to classify individual OC products in the present study. Because of differences in their pharmacological properties, the class of progestin used in a given OC may be relevant to understanding its ability to influence cognitive performance. While all OCs contain a progestin, not all families may have an equal potential to modify scores on the MRT or other cognitive tests, and consequently to cause POC effects.

**Table 2:** The four families of contraceptive progestins and specific brand names represented in the dataset (*N* = 204).

Generation and Family Name	Specific Progestins Used in OCs	Brand Names Represented	Mean Age of Participants (Range)
Generation 1	norethindrone, norethynodrel,	Brevicon 1/35, Brevicon 0.5/35,	*M* = 21.01
(Estranes)	norethindrone acetate,	Demulen 30, Demulen 1/35,	Range = 18-27
*N* = 58	ethynodiol diacetate	Loestrin 1/20, Loestrin 1.5/30,	
		Lolo, Micronor, Minestrin 1/20,	
		Ortho 1/35, Ortho 0.5/35,	
		Ortho 10/11, Ortho 7/7/7	
		Synphasic	
Generation 2	levonorgestrel, dl-norgestrel	Alesse, Aviane, Alysena, Minovral,	*M* = 20.40
(Gonanes)		Portia, Triphasil, Triquilar	Range = 18-30
*N* = 76			
Generation 3	desogestrel, gestodene,	Freya, Linessa, Marvelon, Cyclen,	*M* = 20.39
(Third Generation Gonanes)	norgestimate, etonogestrel,	Tri-Cyclen, Tricyclen-Lo, Tricira Lo,	Range = 18-35
*N* = 48	norelgestromin	Evra*, NuvaRing*	
Generation 4	drospirenone, cyproterone	Yasmin, Yaz, CyEstra, Ginette,	*M* = 20.43
(Spironolactone Derivatives	acetate†	Diane-35	Range = 17-27
and C-21 Progestins)			
*N* = 22			

*Evra (n = 1) and NuvaRing (n = 2) contain third generation progestins but are not administered orally.

†Cyproterone acetate is an old progestin but is included in Gen 4 in the present report because, like drospirenone, it is a potent anti-androgen. It has anti-androgen activity approximately three-fold greater than drospirenone’s (65).

### 3.2 Methods

#### 3.2.1 Participants and description of dataset

Our historical dataset consisted of 204 adult females using various brands of hormonal contraceptives available by prescription in Canada between 1991 and 2015 (*n* = 201 with oral route of administration; see **Table 2** for a list of OC brands used). Our dataset is a compilation of all available OC users tested in studies conducted in our laboratory from 1991-2015, who were using low-dose OCs (35 ug/day EE2 or less). The OC brands present in the dataset are rich and widely varied and represent those commonly used by healthy undergraduates at our institution and how they evolved over 3 decades. Fewer 4^th^ generation OCs are available in the dataset (*n* = 22) than earlier generations of OCs due to the relatively recent introduction of DRSP-based contraceptives, the lesser frequency of their use in North America versus Europe (see 2, 60), and more limited recruitment by our lab for studies involving the MRT over the last 10 years. A criterion for inclusion in the dataset was having performed the 24-item (not 20-item) MRT test of Vandenberg and Kuse (31) as part of an individually supervised study visit to our laboratory, using a time limit of 4-min (not 3-min or 5-min) for each part of the test. All participants performed other cognitive tests too, but because the specifics of those additional tests varied from study to study, depending on the original purpose of the work, only the MRT could feasibly be analyzed here.

Mean age of the participants was *M* = 20.56 (*SD* = 2.32), range = 17-35, with no significant differences across the 4 families of progestins (**Table 2**). Mean daily EE2 dose was matched across the progestin families within 1ug/day, except for the 2^nd^ generation gonanes, which were slightly lower. Except for the ON-OFF study, all data were collected in a blinded fashion. Participants were tested at either the active or inactive phases of the OC cycle. Data acquisition for the 2 phases was always carried out in parallel. Blind testing meant that fewer data from the inactive phase were available, but within each of the contributing datasets the active and inactive phases were matched for the era of their data collection and were thus matched in the compiled dataset as a whole. All participants had an educational level of year 1 of university or higher. Exact details of OC brand names, number of tablets remaining, and any other OC details were collected for each participant during the laboratory visit where the cognitive testing was performed. Although the duration of time on the present OC was not always recorded, average time on OC was ≥4 months where data were available and consequently the pattern of cognitive differences was expected to be stable (pharmacokinetic changes may occur between the first and third cycles of OC use before pharmacologic stability is reached, see 68).

For purposes of the present analysis, data were examined with and without a set of 44 NC controls (non-OC users), described above, who performed the MRT under identical administration and scoring conditions as part of their participation in a study of the MRT and the natural menstrual cycle performed by our lab (21). For all NC controls, phase of the menstrual cycle on the date of cognitive testing was confirmed *via* high-sensitivity radioimmunoassays of 17β-estradiol and progesterone.

The final size of the entire dataset including all groups of participants was 245 individuals.

#### 3.2.2 Statistical analysis

To investigate whether the POC effect replicates across all 4 families of OCs commonly recognized, we performed a factorial ANOVA, with Phase-of-Cycle (active, inactive) and pharmacological family (‘generation’ of progestin) as independent variables, using our archival dataset to test the hypothesis of a generalized POC effect that is associated with OC use and is found pan-generationally. Specifically, we compared accuracies achieved on the MRT during the active and inactive phases of the contraceptive cycle, with women grouped according to the family (class of progestin) to which their OC belonged. A generalized POC effect was predicted to be evident in the ANOVA as a main effect of phase-of-cycle.

Phase of cycle is not the only variable that may influence accuracies on the MRT test. Accuracy may vary across the individual generations of OCs based on differences in their androgenicity. The same ANOVA was also used, therefore, to evaluate a second hypothesis. Wharton et al. (37) was the first to posit that women using 4^th^ generation progestins based on drospirenone (DRSP) might exhibit poorer visuospatial ability (e.g., on mental rotation tests) because of the anti-androgenic qualities of DRSP, which is a partial androgen receptor antagonist (65). In other words, *overall* accuracy on the MRT was predicted to differ systematically across the progestin families as a result of differences in their ability to transactivate androgen receptors (37). Accordingly, our ANOVA was examined to reveal if the different families of OCs showed absolute differences in accuracy on the MRT (i.e., a main effect of progestin family). It should be noted that Wharton’s original findings, which served as the basis for this proposition, were based on a total sample size of only *n* = 7 Yasmin users, raising questions about reproducibility. Two subsequent studies have produced mixed support for Wharton’s hypothesis (44, 60; see also 69). In general, empirical tests have been limited, and the proposed androgen mechanism remains an open question. We analyzed differences between the progestin generations to begin to shed light on the question of mechanism.

### 3.3 Results

#### 3.3.1 Is a POC effect seen uniformly across all families of OCs?

Factorial ANOVA with Phase-of-Cycle and OC Family as between-subjects factors was used to contrast accuracies on the MRT in women tested during the active and inactive phases of the cycle. Only users of *oral* contraceptives were included; transdermal formulations were excluded from the ANOVA. Three outliers scoring below chance on the MRT and one woman whose brand of OC was ambiguous also had to be excluded.

**Figure 3:**
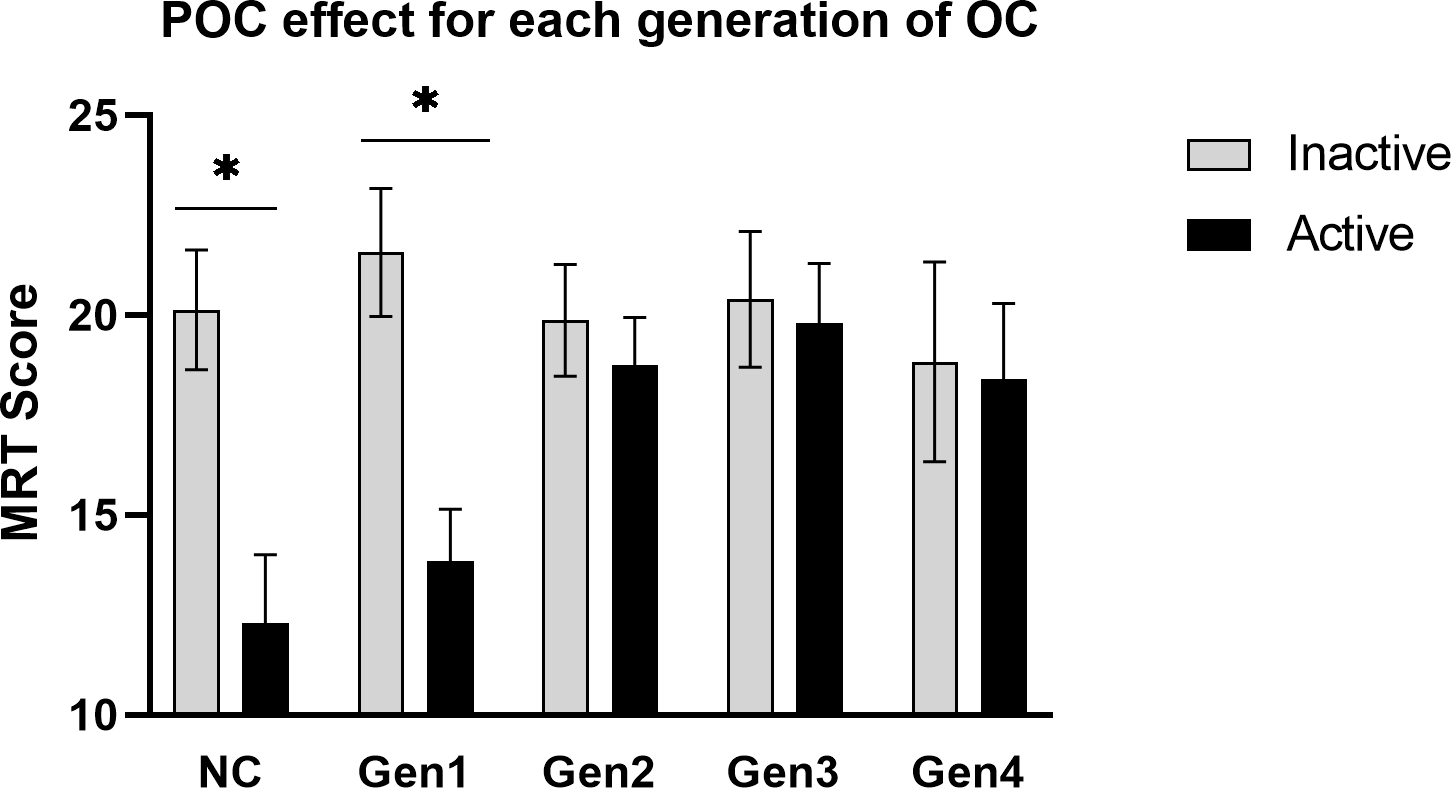
The POC effect for each pharmacological family of progestins. A robust phase-of-cycle (POC) effect was seen among users of early generation OCs (Gen1, n = 55) and naturally-cycling women (NC, n = 44), but to a lesser extent or not at all among the more recent families of progestins (Gen2, n = 74; Gen3, n = 44; Gen 4, n = 22). *p < .01.

The magnitude of the POC effect for each family of contraceptives is displayed in **Figure 3**. Contrary to our hypothesis of a pan-generational effect, a POC effect was identified only for some, but not all, families of OCs. The ANOVA revealed a significant main effect of Phase, *F*(1,228) = 9.77, *p* = .002, whereby accuracy on the MRT was mildly lower in women tested during the active phase of OC use (*M* = 17.55 items correct) than the inactive phase (*M* = 20.41 correct; d = 0.36, across all generations combined). However, the magnitude of the POC effect varied significantly depending on which progestin family was used, Phase x Generation interaction: *F*(4,228) = 2.87, *p* = .024. Tukey *post-hoc* tests revealed that a robust POC effect was identifiable among early generation OC users and among the NC women, but the POC effect decreased progressively in size for more recent families of OCs and was essentially absent among women who used 4^th^ generation progestins (see **Figure 3**).

#### 3.3.2 Effects of the progestin family on MRT scores

**Figure 4:**
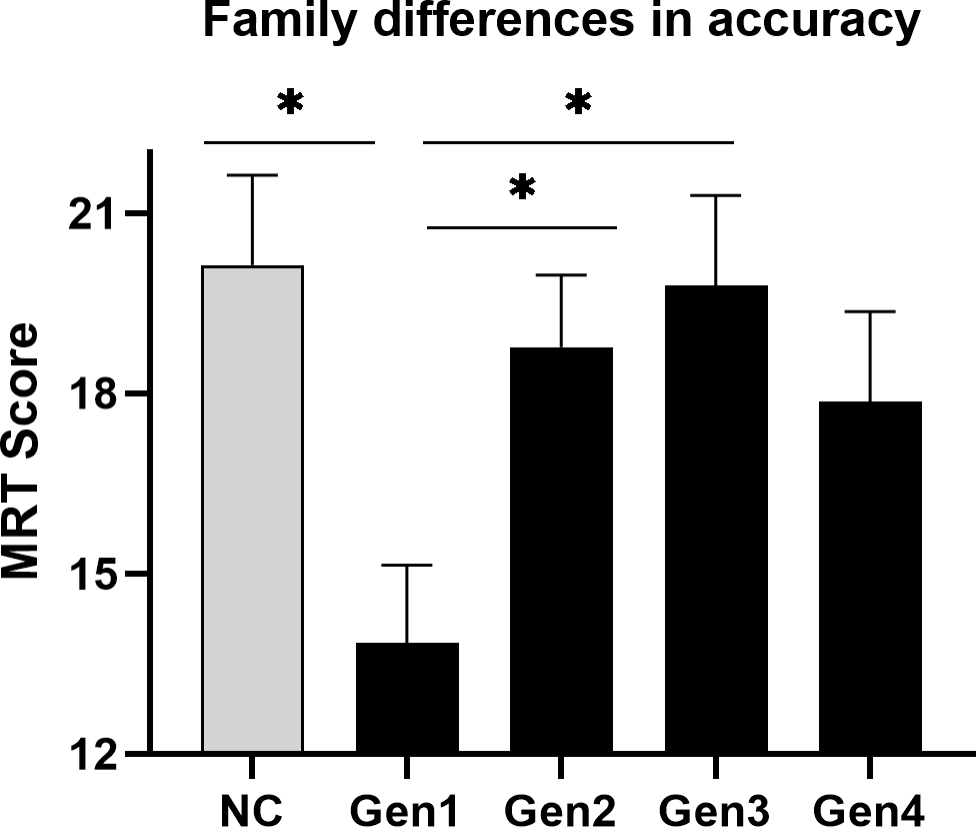
Differences in mean accuracy on the MRT among the progestin families when exogenous steroids were used. During the active phase of the contraceptive cycle (black bars), when OC steroids are taken on a regular daily basis, accuracy of performance on the mental rotation test (MRT) varied significantly across the different families of OCs. The grey bar represents the performance of naturally-cycling controls (NC) tested at menses (inactive phase), which is shown here as a neutral condition. Among OC users, family differences were minimal at the inactive phase (not shown in figure) where no active hormone is used. *p < .05.

Wharton et al.’s hypothesis (2008) predicts that any effect of the progestin family ought to be clearest when exogenous hormones are used *actively*. In light of the Phase x Generation interaction, and to isolate any effect of the progestin families most effectively, a simple effects ANOVA was therefore performed, limiting the analysis to the active phase of the OC cycle only (**Figure 4**). The analysis was run with and without NC controls tested at the *in*active phase to represent a basal level of MRT performance. As shown in **Figure 4**, simple effects revealed a significant effect of the OC family on MRT scores if accuracy was evaluated under conditions of active hormone intake, *F*(4,140) = 3.28, *p* = .013. Tukey-Kramer *post-hoc* tests showed that 1^st^ generation progestins (estranes), even though they possess a moderately high degree of androgenic activity (66), performed significantly worse during OC intake (*M* = 13.84 items correct, *n* = 32) than users of either 2^nd^ (*M* = 18.77, *n* = 43) or 3^rd^ generation progestins (*M* = 19.79, *n* = 24), *p* = .050 and *p* = .036 respectively. Women using 1^st^ generation progestins did not differ significantly from those using 4^th^ generation progestins^1^ (*p* = .321), who likewise had slightly lower accuracies (*M* = 17.86, *n* = 22). No evidence of *superior* accuracy was found in 2^nd^ generation pills containing levonorgestrel (*M* = 18.77, *n* = 43), even though 2^nd^ generation pills are the most highly androgenic OCs of all in terms of their endocrine effects (10). In short, family-dependent differences were observed if OC users were evaluated during active intake, but the pattern of group differences did not support the hypothesis of an effect driven by androgenic properties of the progestins (37).

If the analysis was restricted to monophasic OCs only, or was limited only to OCs containing 30 ug/day of EE2 or higher, the *n*’s were reduced but the pattern of group differences remained unchanged.

### 3.4 Discussion

Contrary to our hypothesis, Study 2 failed to show a POC effect that generalized across all progestin families. Rather, a robust POC effect was found only for Gen1 OCs, replicating the effect found in Study 1 but in a sample twice as large (*n* = 63 versus 134 Gen1 and 2 users, respectively). We also found differences in accuracy among the progestin families, but the differences did not conform to the androgen hypothesis. Differences were visible during active OC use only. If tested during the inactive phase of the OC cycle, when exogenous hormone is not used, accuracy on the MRT was fairly uniform across all OC groups (grey bars, **Figure 3**). During the inactive phase, accuracies in OC users resembled NC women tested at menses, when circulating hormone concentrations are at a nadir. The fact that all groups performed so similarly at the inactive phase suggests that the effects seen for the MRT at the active phase are due to short-term, quickly reversible, effects of OC steroids in the CNS.

Wharton and colleagues (2008) argued that any effect of OCs on mental rotation might reflect their androgenic (or anti-androgenic) properties. Anticipated effects on MRT accuracy would be positive or negative, respectively. This prediction is plausible based on several studies of naturally-cycling (NC) women, which suggest that in non-OC users higher endogenous levels of circulating androgens (e.g., 19, 20) or the experimental administration of testosterone (e.g., 70, 71), is associated with greater accuracy on mental rotation tests. However, some large-sample studies have failed to support any connection between MRT performance and circulating androgen concentrations (e.g., 72). Studies involving OC users are rare and sample sizes are typically small. To our knowledge, Study 2 is the largest to date to test for androgen-related group differences and is the only study to include all 4 families of OC progestins. Our data suggest that androgen activity in the CNS is unlikely to be the major driver of OC family (‘generation’) differences in MRT scores, although it may contribute in a minor capacity to mental rotation along with other variables. Independent of androgens, a separate body of work has suggested that high levels of *estrogens* are associated with lower scores on the MRT in naturally-cycling women (18–21). Because 1^st^ and 2^nd^ generation OCs tend to be higher in estrogenic activity but also higher in androgenic activity, it is unclear how these physiological effects might combine to influence cognitive performance. The question of androgenic versus estrogenic influences as the basis for group differences in the performance of OC users on the MRT was addressed in Study 3.

## 4 Study 3: estrogenic, androgenic, and progestogenic actions of the OC pill

### 4.1 Background and hypothesis

The endocrine effects of OCs vary along several different dimensions simultaneously. Although the OC brands within each progestin family share certain common features, they also exhibit differences that might be relevant to developing a full understanding of their CNS effects. Heterogeneity in the endocrine profile across OC brands is present, both within and between the various pill families. To test whether this heterogeneity is relevant to predicting the mental rotation effect, we followed up Study 2 by systematically coding the endocrine profiles of each of the individual OC brands represented in our dataset. This was done to capture differences that might be relevant to cognitive performance, and to allow further insights into exactly which endocrine characteristics are responsible for the MRT effect.

Individual brands vary in the intensities of their estrogenic, androgenic, and progestogenic actions *in vivo*, depending on dosage differences and which particular forms of estrogen and progestin they contain. Most 1^st^ (and many 2^nd^) generation OCs contain 30-35 ug/day of ethinyl estradiol (EE2), while some OCs developed recently contain lower EE2 concentrations (20-25 ug/day). The estrogenic activity of OCs is not exclusively attributable to their estrogen constituent, however. Some progestins used in OCs (notably the 1^st^ and 2^nd^ generation families) have progestogenic effects, but also exert varying degrees of estrogenic (or sometimes anti-estrogenic) effects (3). Third generation gonanes have no intrinsic estrogen activity. They were developed to minimize androgenic side-effects associated with the 2^nd^ generation progestins, which in turn are more highly androgenic than the moderate intensity estranes (1^st^ generation progestins). Recently, 4^th^ generation progestins, notably DRSP, have been developed and are purer progestogens, devoid of androgen activity (73). Some of the newer progestins are potent progestogens despite their absence of androgenic effects and possess several-fold greater progestogenic effectiveness than progesterone itself (65). The progestin doses actually used in OCs are reduced accordingly (3). Some progestins (e.g., cyproterone acetate, dienogest, drospirenone) demonstrate anti-androgenic activity at the concentrations used in OCs (65). Based on these complexities, many OCs simultaneously possess estrogenic, androgenic, and progestogenic actions in terms of their biological actions in tissue, and exert each of these effects to varying degrees depending on exactly which brand of OC and which family of progestin we consider. Any of these biological activities or some combination of them might explain the observed effects of OCs on MRT performance.

There have been at least 2 previous attempts to formally investigate the hormonal underpinnings of the visuospatial effect in women using OCs. In a group of 56 OC users, all of whom were using 1^st^ or 2^nd^ generation OCs and were tested during the active phase of the OC cycle, Hampson and Moffat (38) reported that the overall estrogenic potencies of the OCs used by women in their sample were a significant inverse predictor of performance on 2 visuospatial tests. Relative potencies had been established by bioassays or receptor studies (74; see 38) and reflected the combined estrogenic effect of each brand of OC attributable jointly to both its estrogen and progestin constituents. Using an alternative approach and a larger sample size, Beltz etal. (35) found that the nominal EE2 dose in a group of OC users significantly predicted scores on the MRT (β = -.26). Importantly, in the Beltz et al. study (2015), OC users were analyzed as a combined group without considering POC effects. Thus, OC users were included in the statistical analysis irrespective of whether they had been tested on the MRT at the active or inactive phase of the OC cycle. Accordingly, Beltz’s study may underestimate the true magnitude of the estrogen effect. In the present analysis, we adopted the approach advocated by Hampson and Moffat (38). Thus, biological potency not face dosage was used as an index of hormone action.

### 4.2 Methods

#### 4.2.1 Coding of OC brands

For each woman in our dataset, the specific brand of OC pill being used was independently coded for its estrogenic, androgenic, and progestogenic properties by using standard tables of the relative potencies of OCs available in Dickey and Seymour (67) or earlier editions of the same source (e.g. 74). Numerical values assigned by Dickey to each specific OC brand are data-driven and based on *in vivo* bioassays or receptor studies (e.g. 75) derived from humans or from pre-clinical investigations of laboratory animals. As such, they are an objective quantification of each brand’s capacity to exert biological effects. The estrogenic activity of each brand of OC is expressed relative to pure ethinyl estradiol, the androgenic activity of each brand is expressed relative to methyltestosterone, and progestogenic activity is expressed relative to norethindrone (67). Importantly, this method of coding considers the total bioactivity of each OC brand integrated over a 28-day window relative to its index compound (e.g., methyltestosterone) and, in contrast to classifications based on dosage alone, accounts for not only differences across brands in dose administered but also differences in the intrinsic biological strengths of the individual progestins and any incremental estrogenic or androgenic actions attributable to each OC’s progestin component. It therefore affords a more refined and accurate picture of true differences in the endocrine effects of different OC formulations than dose considered alone. In effect, this endocrine coding method places all OC brands on a common measurement scale, allowing biological differences among OCs to be reflected irrespective of the identities of the specific progestins that comprise each brand. Relative to straight dosage-based comparisons, it offers greater precision, corresponds more closely to clinical observations (75), can encompass multiphasic as well as monophasic OC formulations, and avoids the need to entertain only small homogeneous groupings involving a single progestin when comparing different brands of OCs (*cf*. 35). Although some inexactness is still acknowledged (67, 75), part of which stems from individual variation in women’s metabolism of the contraceptive steroids (7, but see 8), average differences between brands on the dimensions of estrogenicity, androgenicity, and progestogenicity are captured best by bioassay data making it a superior choice as an index of true differences between OCs in their capacity to exert endocrine effects *in vivo*.

Accordingly, each OC brand used by the 200 women in our dataset was assigned 3 independent ratings based on Dickey and Seymour (67) or related sources (74), reflecting its estimated estrogenic, progestogenic, and androgenic actions *in vivo*. Users of 2 OCs in our dataset, namely one 4^th^ generation (Diane-35; alternatively CyEstra, *n* = 5) and one 1^st^ generation OC (Demulen 30, *n* = 5) could not be coded, because these brands of OCs are not approved for use in the USA and thus no endocrine ratings for them were available in Dickey and Seymour (67).

#### 4.2.2 Statistical analysis

Multiple linear regression with forced entry was used to investigate whether the estrogenic, androgenic, or progestogenic actions of OCs are significant predictors of accuracy on the MRT, and to evaluate their relative importance. The total MRT score of each participant was entered as a dependent variable, and the estrogenic, progestogenic, and androgenic ratings for each specific brand of OC from Dickey and Seymour (67) were used as predictor variables. In our sample, age was not significantly correlated with MRT scores (*r* = .03), likely reflecting the narrow age range of the present dataset and the fact that the MRT shows negligible age-related changes in young adulthood. Thus age was not entered as a predictor variable in the regressions. Progestogenic potency of each OC was included as a predictor for completeness, despite a lack of evidence based on previous literature to suggest a progestogen-based effect on MRT performance in either naturally-cycling women (e.g., 18–21) or in the only previous study of OCs to address this question (35). The OC ratings of progestogenic potency were skewed and were log-transformed prior to analysis to reduce skewness. Two outliers who had a total score ≤3 on the MRT (out of a possible maximum score of 48) were excluded when performing the regressions.

Only women evaluated at the active phase of the OC cycle were included in the regression analysis (*n* = 104). A matching regression performed for OC users evaluated on the MRT at the inactive phase can be found in the Supplementary Materials (**Table S3**).

### 4.3 Results

**Figure 5:**
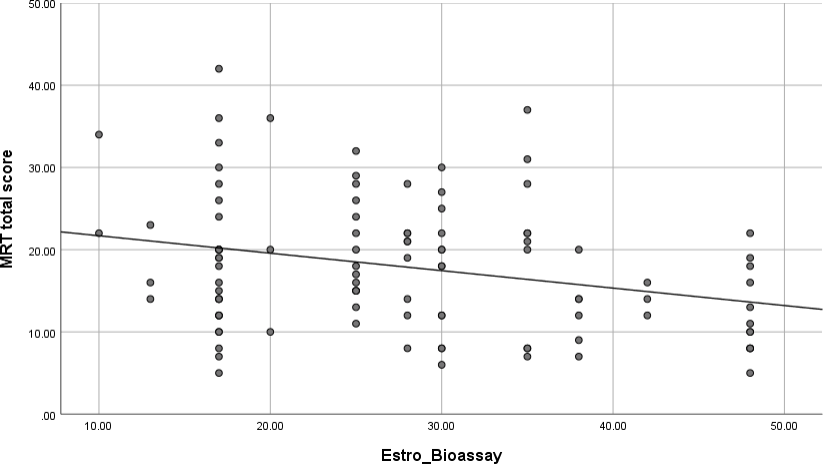
Scatterplot of the zero-order correlation (with best-fitting regression line) between overall accuracy on the Mental Rotation Test (MRT) and the estrogenic potencies of the oral contraceptives (OCs) used in our sample. All OC users were tested during the active pill ingestion phase of the contraceptive cycle (n = 104). OCs higher in estrogen potency were associated with poorer spatial accuracy on the MRT test. Estrogenic potencies for each contraceptive brand were obtained from tables in Dickey and Seymour (67), *Managing contraceptive pill patients and other hormonal contraceptives* (17^th^ ed) or earlier editions of the same source, and are based on bioassays or receptor studies. Estro_Bioassay = relative estrogen activity of each OC brand.

Forced-entry regression revealed that the endocrine profile of the OC pills significantly predicted scores achieved on the MRT, *F*(3,99) = 3.28, *p* = .024. The regression model is summarized in **Table 3** (top). Individually, only an OC’s estrogenic potency, but not its androgenic or progestogenic potency, was a significant predictor of accuracy (β = -.30, *p* = .004). OCs higher in biological estrogen activity were associated with lower scores on the MRT, irrespective of their nominal EE2 dosage. The relationship is shown in **Figure 5** as a simple scatterplot with line of best fit. This outcome agrees with the results of Hampson and Moffat (38) who found that higher estrogen potency of OC brands predicted poorer scores on a different but related visuospatial test, Space Relations from the Differential Aptitude Test (76). It also agrees with Beltz et al. (35) who identified a negative association between EE2 dose and accuracy on the MRT, based on differences in nominal OC dosage instead of overall biological activity as indicated by *in vivo* pharmacological studies (67). In support of these findings, high estradiol phases of the menstrual cycle are reportedly associated with lower accuracies on the MRT among naturally-cycling women (e.g., 18–21, 33, 42).

**Table 3:** Results of the multiple regression analyses.

	R	R^2^	F	Predictor	Beta	t, *p*-value
OC Users at Active Phase (*n* = 104)	.30	.09	3.28*	Estro	-.30**	-2.93, *p* = .004
				Andro	-.08	-0.77, *p* = .443
				Progest	-.08	-0.80, *p* = .423
Generations 1 and 2 Only (*n* = 70)	.39	.15	3.88*	Estro	-.40**	-3.30, *p* = .002
				Andro	-.13	-0.95, *p* = .346
				Progest	.10	0.74, *p* = .461

Dependent variable = Total MRT score (max = 48). Estro, estrogenic potency; Andro, androgenic potency; Progest, progestogenic potency (log-transformed).

*p < .05, **p < .01.

Current OCs that contain 3^rd^ and 4^th^ generation families of progestins possess limited or no androgenic activity, and often have only weak estrogen effects due to reduced doses of EE2 in a number of the 3^rd^ and 4^th^ generation formulations (e.g., Yaz = 20 ug/day). Re-running the multiple regression with only the 1^st^ and 2^nd^ generation OCs included (*n* = 70) produced an acceptable range in both the estrogenic (range = 10 to 48) and androgenic potencies (range = .17 to .80) and increased the magnitude of the predictive relationships observed (**Table 3**, bottom). Here, too, only differences in estrogen potency across OC brands were a significant predictor of accuracies on the MRT, β = -.40, *p* = .002 (**Table 3**).

## 5 General discussion

Mental rotation is a basic visuospatial ability used in science, technology, construction, and engineering disciplines that require the accurate visualization of spatial relationships among objects, parts of objects, or the visualization of movements in three-dimensional space. In the present work, we analyzed an archival dataset of OC users who were tested on a standardized test of mental rotation, the MRT (31). With an overall sample size of 201 OC users it is, to our knowledge, the largest sample to date to address the question of whether mental rotation is influenced by the use of OCs. We found a significant POC effect—a difference between average accuracies on the MRT during active pill usage compared with a baseline condition where no active hormone was being used. Accuracy was higher under the no-use (“inactive”) conditions. The POC effect was seen prominently in women using early-generation OC pills, was attenuated in users of second-generation progestins, and was virtually absent among women taking current, third-generation, contraceptives. Changes in the hormonal constituents of OCs over time likely explain these generational effects. In particular, regression of MRT scores on the estrogenic, androgenic, and progestogenic biopotencies of a wide range of OC brands across 4 generations of OC pill formulations revealed that the capacity of an OC to produce estrogenic effects in tissue is one variable which contributes to individual differences in visuospatial performance.

Our findings reinforce cognitive differences seen over the menstrual cycle in naturally-cycling women, although those differences like the ones seen here are often subtle. During the past 3 decades, studies of NC women (not using OCs) have reported that performance on the MRT is modestly reduced at phases of the ovarian cycle characterized by high levels of circulating 17β-estradiol (see 41, for a recent review). A negative correlation between individuals’ scores on the MRT and current estradiol concentrations in serum or saliva has been shown (e.g., 18–21). Using a different paradigm, oral contraceptive use, the present data converge on the notion that reproductive steroids have subtle effects on specific visuospatial functions and extend these observations to the synthetic steroids that are used in OCs.

Our data are among the first to suggest a POC effect in OC users. They extend early findings by Moody (33) and Silverman and Phillips (34). While those early studies had significant methodological shortcomings that rendered them inconclusive, they helped to inform the POC hypothesis tested in the present report. Even earlier data from our own lab (77), using an independent dataset not included in the present report found that OC users assessed at the active phase of the OC cycle differed significantly from NC women assessed during menses on several other sex-differentiated cognitive tasks (that did not include the MRT) and concluded that ‘functionally high levels’ of hormones present during OC use might have implications for certain dimensions of cognitive performance.

Although mental rotation was our major focus, it was not the only cognitive function to show a POC effect in the present work. Study 1 also revealed a phase-of-cycle effect on rapid motor learning and execution and on word fluency. Unlike the MRT, which exhibits a well-established performance advantage in favor of males, fluency and motor learning exhibit sex differences in favour of *females* in the general population (78, 79). Relative to the MRT, these tasks showed an opposite, reversed, direction of POC effect in the present study, i.e. improvement during the *active* phase of the contraceptive cycle. This too is consistent with studies of naturally-cycling women showing similar functional selectivity in the cognitive effects seen under high 17β-estradiol conditions (e.g., 18). In OC users, studies investigating non-spatial facets of cognition are infrequent. Almost all have focused narrowly on declarative memory (but see 26, 69). This highlights the novelty of the present findings, which go beyond mental rotation.

Insofar as data are available, our results are compatible with POC effects identified in past studies of OC users on non-spatial tasks. Mordecai etal. (26) found improved verbal memory during the active pill phase in a study using a within-subjects design. Likewise, Hampson (25) found improved working memory during active usage. Verbal fluency and motor learning, however, have rarely been studied. In the present data, all effects seen under active OC use, including the MRT, are in directional agreement with effects on cognitive functions identified under high estradiol conditions in naturally-cycling women. The fact that similar effects are observed under active OC use suggests that exogenous hormone intake *via* the OC pill is the agent likely responsible for these cognitive effects. While OCs also inhibit the *endogenous* production of ovarian steroids, this inhibition is not quick to resolve. In most OC users, endogenous concentrations remain at very low early follicular values during the 7-day inactive phase, i.e. they closely resemble the menstrual phase concentrations of naturally-cycling women, where ovarian activity is negligible (e.g., 12, 26, 44). The suppression of endogenous production, because it spans across both the active and inactive phases, does not offer a satisfactory explanation for the POC effects we observed over the OC cycle, particularly their time course, which includes a rapid dissolution of the effects in the inactive phase followed by their re-appearance upon the return of exogenous steroid intake.

Several caveats must be noted. Though it is an implausible mechanism to explain the cognitive effects seen here, the inhibition of endogenous hormones by OCs might nevertheless contribute to other phenomena associated with long-term OC use such as, for example, incremental reductions in bone density that accrue under long-term exposure (80). Such a possibility is not ruled out by the present findings. Secondly, the cognitive functions we studied here were intentionally selected because they are known to exhibit differences in mean performance as a function of an individual’s biological sex, and there is reason to believe they may be sensitive to circulating hormones (see 81). However, many if not most cognitive functions are not sexually differentiated. Accordingly, they might be expected to show no changes in response to OC use based on these same considerations. The present findings illustrate that OC-mediated effects on CNS functions are possible and offer ‘proof of principle’, but further research will be needed to define which cognitive functions more generally are influenced by OCs and which are not, and the present findings should not be overgeneralized. Revealing the breadth and limits of the POC phenomenon is an important objective for future research endeavors. Thirdly, the fact that we saw a perceptible difference in mental rotation performance when exogenous hormones were higher does not imply OCs produce ‘supra-physiological’ concentrations, as is sometimes claimed, or that they even reach the working concentrations attained during a natural menstrual cycle by their endogenous analogs. Indeed, direct assays of EE2 concentrations and their 24-hr time course following active pill ingestion (e.g., 82, 83), as well as the diminished magnitude of the POC effect we observed in users of contemporary OCs that contain lower doses of EE2, suggest contemporary OCs have physiological effects that are fairly modest.

The diminishing POC effect we saw over recent generations of OCs is a novel finding. It is unlikely to be explained by cohort effects, because all families of OCs in our dataset showed similar mean performance on the MRT if they were tested during the inactive phase of the OC cycle (**Figure 3**). Accuracies matched those of non-users tested at the same phase, when ovarian production of hormones is quiescent. Progestin family differences were seen only among women tested during the active phase of intake (which was always evaluated concurrently with the inactive phase during data collection). We propose instead that a diminished POC effect and generally higher levels of MRT performance during the active phase for more recent than older OCs reflects changes in the hormonal constituents of OCs over the past 3 decades. These include reductions in EE2 dose and associated changes in progestins, or a shortened inactive phase (e.g., Yaz, Loestrin 24) where exogenous washout normally occurs, or other changes that attenuate endocrine differences between the active and inactive phases of the OC cycle. If POC effects vary by generation it might explain inconsistencies in past literature regarding OC effects on cognition.

One popular theory speculates that effects of OCs on mental rotation are attributable to their progestin constituent, specifically the extent to which an OC exerts androgenic or anti-androgenic effects *in vivo* (37; or see also 84). The present dataset failed to support the androgen hypothesis, either in terms of the group differences found across the different families or in our multiple regression analyses where androgen activity was taken into account on a brand-by-brand basis. Specifically, androgen potency was not a significant contributor to MRT scores in our regressions, and the slightly diminished MRT score seen among 4^th^ generation users was so slight as to be non-significant.

Lack of support for the androgen hypothesis is not altogether surprising. Androgenic effects of OCs are very weak compared to testosterone itself, making it less likely that effects on cognition would be perceptible. This hypothesis is still largely speculative and there is a lack of empirical support for it more broadly. Wharton et al (37) result showing lower accuracy on the MRT in Yasmin users (*n* = 7) was not replicated by Griksiene and Ruksenas (44), who found no differences between 3^rd^ (*n* = 10) and 4^th^ generation OCs (*n* = 11) on a mental rotation test. A later study found that women taking anti-androgenic OCs (*n* = 35) were less accurate at mental rotation than naturally-cycling women, but other generations of progestins were not evaluated and thus no true generational effect was actually demonstrated (60). Recently, Gurvich et al. (69) found no significant POC effect on a different type of visuospatial task requiring recall of a learned route through a grid, but anti-androgenic OCs (*n* = 17) did perform more poorly than OCs containing levonorgestrel (*n* = 18), an androgenic progestin.

Our study is the first to evaluate androgenicity in a dataset where all 4 historic families of OC progestins were represented, enabling us to see a fuller picture. It is worth re-emphasizing that progestins in the present study were classified by their chemical structure not by the timing of their introduction into the marketplace. Thus our Gen1 and Gen2 families included OC formulations that are still available, albeit used infrequently by women today. For example, Alesse and Minovral were both considered 2^nd^ generation OCs in the present work because they both contain levonorgestrel, but were introduced at very different times. Only 50% of the Gen1 brands listed in **Table 3** are still marketed. For these reasons, our dataset is of scientific value but POC effects may be less applicable to many OC users at a clinical level today. Studies that include only the 2 most recent OC generations do not evaluate androgenicity across its entire range. While we believe that classification based on progestin identities is the most theoretically defensible, it is possible that different results would be obtained if brands of OCs were classified into generations based on market timing instead, which is an alternative basis for classifying progestins sometimes used in epidemiologic studies (63).

While POC effects may be less likely, current brands still vary considerably in their estrogen potency and our data suggest that pill estrogenicity may be the primary contributor to OC-related differences in performance on the MRT. It may also underlie the differentially large POC effect seen for the highly estrogenic Gen1 OCs. In our regressions, we used an empirically-derived coding scheme (67) based on published evidence from bioassay tests (e.g., actions in the rat ventral prostate relative to methyltestosterone; 85) to quantify the estrogenic, androgenic, and progestogenic effectiveness of each individual brand of OC pill. This method of quantifying bioactivity avoids the pitfalls of trying to make comparisons across OC brands based on dosage alone. A given brand may have higher or lower estrogen activity than its nominal EE2 dose implies, depending on which exact progestin the EE2 is paired with (e.g., see Ortho 0.5/35; 74). Progestogenic potency was also coded, but the progestogenic activity of the OCs in our dataset was found to be highly skewed. Although corrected by log-transformation, we consider our progestogen results to be only provisional and further investigation in future work is recommended. On the other hand, our regressions did support an estrogen-driven effect of OCs on women’s MRT scores. OC brands having greater estrogenic effects were associated with lower accuracies on the MRT (**Figure 5**), irrespective of generation. This pattern was most pronounced among OCs possessing greater levels of estrogenicity, despite the higher androgenicity that is also associated with 1^st^ and 2^nd^ generation pills. Our study is the first to assess all 3 biological effects simultaneously, allowing their independent effects on spatial ability to be identified. Our findings demonstrate the relevance of estrogen and converge with similar conclusions by two previous studies (35, 38) that used smaller less diverse samples and implicated the estrogen activity of OCs in spatial cognition by utilizing alternative methods. In our dataset, Gen1 OCs were significantly higher in estrogenic activity than the other Gens as demonstrated by objective bioassay tests, which might explain the lower MRT accuracies seen for Gen1 OCs during the active phase of pill ingestion and the larger POC effect in Gen1 including significant ‘bounceback’ in MRT performance during the inactive phase.

An effect on the MRT that is mediated by estrogen is plausible given similar effects reported for 17β-estradiol in naturally-cycling women (e.g. 18, 20, 21). Little is known of ethinyl estradiol’s sites of action in the CNS, but ERα and ERβ are expressed regionally in several regions of the CNS that are important in cognitive functioning (14, 86, 87) and EE2 is able to bind to intracellular ERs. In fact, EE2 displays an affinity for the ERα receptor even greater than the endogenous ligand, 17β-estradiol (88). Following oral ingestion of an active OC pill, serum concentrations of EE2 peak approximately 1-2 hrs later and may transiently reach mid-follicular values before declining to early follicular levels until the next pill is taken 24-hr later (82, 83). EE2 concentrations seen at peak vary depending on the EE2 content of the OC pill that is taken (52, 82) and on individual differences in absorption and metabolism (7; but see 8). Although the peak concentration is not sustained for long and typically drops to low basal levels within just a couple of hours, genomic effects initiated by the initial EE2 availability in the bloodstream might be longer-lasting. The time course of the effects initiated is not presently known. The natural ligand, 17β-estradiol, has been shown in laboratory animals to exert a range of effects on neurotransmitter synthesis, release, and metabolism (14) through binding to estrogen response elements at acceptor sites on the nuclear DNA and influencing the transcription of target genes. Estradiol also exerts rapid membrane-associated effects in neurons, as well as effects on synaptic architecture in responsive brain regions (15). It remains to be confirmed if the synthetic estrogen EE2 has similar effects at the neuronal level. Recent functional imaging studies have reported differences in regional brain activity and connectivity in OC users compared with women not using OCs (11), suggesting a potential for cognitive/behavioral outcomes to be affected by OC use, but few studies have looked at outcomes in terms of overt behavioral changes that might be relevant to women’s actual day-to-day functioning. Not all OCs may be equally capable of exerting CNS effects. Average estrogen doses have been lowered from 150ug in 1960 to as little as 10-15 ug/day today. It is possible that cognitive effects only occur in conjunction with OCs having high enough doses of EE2 to produce an impact on the brain circuitry that underlies cognitive function.

The present report has limitations, but also several strengths. We used prospective recruitment and assignment to conditions in Study 1. Studies 2 and 3 relied on retrospective data. As such, the present study cannot conclusively establish causation because our design is purely observational. Accordingly, a prospective placebo-controlled design would be desirable in a future study. A placebo control is difficult, however, where contraception is the topic of investigation due to ethical issues surrounding assignment of fertile women to a placebo condition. Although prone to practice effects, future work on cognitive function might also consider using a within-subjects design where cognition is evaluated in OC users before and after the onset of treatment. To capture changes in OC formulations over a 30-year time window, as in Study 2 or 3, a between-subjects design inevitably must be used. Strengths of the present work include the broad temporal scale it covers; use of a consistent, trusted and standardized spatial test; and data collection that was performed in parallel for both the active and inactive phases of the cycle in all ‘eras’ covered by our 30-year window. Although societal changes occurred over this interval, differences in average scores on the MRT for the progestin families were seen only at the active (not the inactive) phase of the OC cycle. Steady levels of accuracy at the inactive phase over the 30 years of data collection argues against societal changes in educational practices, gendered play, or unknown social changes as explanations for the family-wise differences we observed. The fact that we found better not worse performance on the MRT during the inactive (menstrual) phase of the cycle would similarly argue against negative stereotypes related to menstruation as the basis for a POC effect.

## 6 Conclusions

The present study reinforces and further validates studies of NC women reporting an effect of estradiol on spatial functions. Our data suggest generalizability to ethinyl estradiol, the synthetic form of estradiol used in nearly all current OCs. Lower estrogen potency was associated with better spatial performance. Effects tended to be modest in size and are not of clinical concern. These findings advance our emerging understanding of OC effects in the human CNS and at a broader level the associations between reproductive steroids more generally and female brain function. Knowledge of such effects can help promote more informed decision-making on the part of OC users and their healthcare providers. Our use of an endocrine coding scheme that reflects the biological effectiveness of OCs *in vivo* and not just face dosage can be applied in future research studies, in order to promote a deeper and more accurate analysis of OC effects in behavioral investigations.

## Data availability statement

The raw data supporting the conclusions of this article will be made available by the authors, without undue reservation.

## Ethics statement

The studies involving human participants were reviewed and approved by the Non-Medical Research Ethics Board, University of Western Ontario, London, Ontario, Canada. The patients/participants provided their written informed consent to participate.

## Author contributions

EH was responsible for study conception, study design, and data interpretation. KE contributed to study design, data acquisition, and preliminary scoring. EM contributed to data acquisition and scoring. CF contributed to data compilation, data checking, and literature search. Statistical analysis was performed by EH, who wrote the first draft of the manuscript. All authors had an opportunity to contribute to the interpretation of analyses and manuscript revision, and have approved the final version. EH was responsible for funding acquisition and resources, and for student supervision. All authors contributed to the article and approved the submitted version.

## Acknowledgments

The authors wish to gratefully acknowledge the following undergraduate trainees who contributed to data collection: L. Blumenthal, J. Finestone, M. Iqbal, N. Levy, C. Szekely, E. Wolff. This work was funded by the Natural Sciences and Engineering Research Council of Canada (Grant No. #138017, #2015-03662, and #2020-04989 to Dr. E. Hampson). Dr. Hampson was supported by a Senior Career Research Chair from the Canadian Institutes of Health Research.

## Conflict of Interest

The authors declare that the research was conducted in the absence of any commercial or financial relationships that could be construed as a potential conflict of interest.

## Publisher’s note

All claims expressed in this article are solely those of the authors and do not necessarily represent those of their affiliated organizations, or those of the publisher, the editors and the reviewers. Any product that may be evaluated in this article, or claim that may be made by its manufacturer, is not guaranteed or endorsed by the publisher.

## Footnotes

1 Because the 4^th^ generation OCs showed no evidence of a phase-of-cycle effect, we ran an analysis with all 22 4^th^ generation users included, to bolster the limited sample size available for the 4^th^ generation group and maximize the likelihood that any group difference would be detected.
