# Supplementary material for: Effects of oral contraceptives on spatial cognition depend on pharmacological properties and phase of the contraceptive cycle
Source: Front Endocrinol (Lausanne). 2022 Sep 6;13:888510. doi: 10.3389/fendo.2022.888510 (PMC9487179; doi:10.3389/fendo.2022.888510)
Supplement: Supplementary file 1 [file Table_1.docx]

**Supplementary Materials**

**Table S1.** Brand names of the oral contraceptives used by participants in Study 1 (*N* = 63)

_____________________________________________________________________________________

*Monophasic OCs No. of Participants Name of Progestin Nominal EE2 Dose (ug)*

Ortho 1/35 5 norethindrone 35

Ortho 0.5/35 2 norethindrone 35

Demulen 30 7 ethynodiol diacetate 30

Brevicon 0.5/35 2 norethindone 35

Minovral 7 levonorgestrel 30

Loestrin 1.5/30 1 norethindrone acetate 30

*Multiphasic OCs*

Ortho 7/7/7 20 norethindrone 35

Triphasil 13 levonorgestrel 30/40/30

Synphasic 5 norethindrone 35

Triquilar 1 levonorgestel 30/40/30

____________________________________________________________________________________

**Table S2.** Cognitive and motor tests used in the ON-OFF study

***Visuospatial Tests***

Mental Rotations Test Participants are asked to identify which two out of four forced-choice alternatives are rotated depictions of a

Source: Vandenberg & Kuse, 1978 3D target object. The incorrect objects superficially resemble the target but cannot be rotated into alignment.

24 items, 4 minutes allowed for each of the two halves of the test. Score is the number correct, after applying a statistical correction for guessing. Max score = 48

Paper Folding Test Participants are asked to identify which of five choices correctly shows the location of the holes in a piece of paper

Source: Educational Testing Service that has been folded, punched, and then unfolded. 20 items, 6 minutes allowed. Score is the number correct,

(Ekstrom et al., 1976) after statistical correction for guessing. Max score = 20

***Verbal Fluency Tests***

Oral Fluency Participants are asked to generate aloud as many words as possible that begin with a specified letter of the

Source: Benton, 1968 alphabet, using a one-minute time limit for production. The score is the number of unique words generated.

Controlled Associations Test Participants are asked to generate as many words as possible that are the same or closely similar in meaning to

Source: Educational Testing Service target words specified by the experimenter. Twelve minutes are allowed to generate lists for 8 different target

(Ekstrom et al., 1976) words. The number of acceptable synonyms generated was independently tabulated by two raters. Inter-rater

reliability was high (*r* = .90, *p* < .001)

***Motor Learning and Coordination***

Manual Sequence Box – Acquisition A simple three-step sequence of movements is demonstrated, consisting of pushing a button, pulling a lever, and

Source: Kimura, 1977 then pressing down on a bar using a required hand orientation to perform each step. The time (in sec) needed to

reach a specified learning criterion is recorded.

Manual Sequence Box – Execution The same pattern of movements described above is performed under speeded conditions. The time required (in

Source: Kimura, 1977 sec) to perform 10 consecutive sequences without error is recorded. The execution condition always followed the

acquisition stage of the task.

**Table S3.** Supplementary multiple regression analysis (Study 3)

______________________________________________________________________________________________________________________

**R R^2^ F Predictor Beta t, *p*-value**

OC Users at Inactive Phase (*n* = 76) .13 .02 0.44 Estro .12 0.90, *p* = .370

Andro .08 0.68, *p* = .501

Progest -.10 -0.78, *p* = .441

______________________________________________________________________________________________________________________

Dependent variable = Total MRT score (max = 48). Estro = estrogenic potency; Andro = androgenic potency; Progest = progestogenic potency (log-transformed)

**p* < .05 ***p* < .01

Notes on Table S3:

Major results of the multiple regression analyses are reported in the Results section of the main article (Section 4.3 and Table 3, see main article).

In Table S3, shown here, only women assessed on the Mental Rotations Test (MRT) during the inactive phase of the OC cycle were included in a linear regression analysis. The women included here were tested during menses, when active hormone is not being ingested from any exogenous source. The results of this supplemental analysis failed to demonstrate any significant predictive relationships whatsoever between the hormonal characteristics of a woman’s OC pill and MRT scores achieved during the inactive phase of the OC cycle, *F*(3,71) = 0.44, *p* = .729.

This outcome is consistent with the mechanism we have proposed. Namely, we propose that the OC effects observed during the active phase of the contraceptive cycle (see main article) are associated with the active intake of exogenous steroids that is occurring at that time.
